# Prevalence and determinants of primary dysmenorrhea among rural adolescent girls: A cross-sectional descriptive study

**DOI:** 10.6026/973206300213527

**Published:** 2025-10-31

**Authors:** Patel Divyanka Navinbhai, Mahalakshmi B., Siva Subramanian N.

**Affiliations:** 1 Department of Obstetrics and Gynecological Nursing, Nootan College of Nursing, Sankalchand Patel University, Visnagar, Gujarat - 384315, India; 2 Department of Pediatric Nursing, Nootan College of Nursing, Sankalchand Patel University, Visnagar, Gujarat - 384315, India; 3 Department of Psychiatric Nursing, Nootan College of Nursing, Sankalchand Patel University, Visnagar, Gujarat - 384315, India

**Keywords:** Primary dysmenorrhea, adolescent girls, prevalence, determinants, body mass index

## Abstract

Primary dysmenorrhea is a common cause of morbidity among adolescent girls, impacting school attendance and quality of life. This community-based cross-sectional study was conducted among 300 rural adolescent girls (13-18 years) in Dadra and Nagar Haveli using a structured interview schedule and Numerical Pain Rating Scale to assess prevalence and determinants of dysmenorrhea. Data showed that 61.3% experienced moderate pain and 27% severe pain, with significant associations between severity and BMI (p = 0.031), family history (p = 0.015) and family type (p = 0.029). No significant associations were observed with age, menarcheal age, cycle frequency, flow duration, religion, or income.

## Background:

Primary dysmenorrhea is defined as painful menstruation occurring in the absence of pelvic pathology and is one of the most common gynecological problems among adolescent girls [1]. The pain is typically cramp-like, localized in the lower abdomen and may radiate to the back and thighs [2]. It is often accompanied by systemic symptoms such as nausea, headache, fatigue and irritability [3]. Studies have highlighted that dysmenorrhea is a leading cause of recurrent absenteeism from school, decreased participation in academic and social activities and reduced quality of life during adolescence [4]. Globally, the prevalence of primary dysmenorrhea has been reported to range from 50% to over 90%, depending on the population studied, diagnostic criteria and severity definitions [5]. A meta-analysis of student populations revealed a pooled prevalence of approximately 66%, suggesting that two-thirds of adolescent girls are affected by some degree of menstrual pain [6]. In India, several studies have reported prevalence rates ranging from 50% to 80%, with a substantial proportion of girls experiencing moderate-to-severe pain [7]. Clinical determinants include early age at menarche, prolonged menstrual flow, heavy bleeding, irregular cycles and positive family history of dysmenorrhea [8]. Therefore, it is of interest to describe the prevalence and determinants of primary dysmenorrhea among rural adolescent girls.

## Methodology:

## Study design and setting:

A community-based cross-sectional descriptive study was conducted in selected rural areas of Dadra and Nagar Haveli to assess prevalence and determinants of primary dysmenorrhea among adolescent girls.

## Population and sampling:

Adolescent girls aged 13-18 years with regular cycles and no pelvic pathology was included. Using convenience sampling, 300 participants were recruited after obtaining school permission, parental consent and participant assent. Sample size was calculated assuming 70% prevalence, 95% confidence level and 5% error.

## Data collection:

A validated structured interview schedule was used to collect socio-demographic data, clinical variables and pain characteristics. Severity was measured using the Numerical Pain Rating Scale and associated symptoms were scored on a five-point Likert scale. Interviews lasted 20-25 minutes and were conducted privately.

## Data analysis:

Data were entered in SPSS v25. Descriptive statistics summarized findings and chi-square (χ^2^) test assessed association between severity and selected variables (p < 0.05).

## Ethical considerations:

Institutional ethics approval and school permissions were obtained. Confidentiality was maintained and girls with severe pain were referred for clinical care.

## Results:

Table 1 (see PDF) shows that most participants were aged 14-15 years (54.3%), Hindu (81.7%) and from rural areas (68.3%). Two-thirds belonged to nuclear families and nearly half had monthly income below ₹5000, reflecting a lower-socioeconomic profile. Most girls attained menarche after 12 years (77.3%), had regular 26-28 day cycles (56.0%), 5-7 days of menstrual flow (68.3%) and used 4-5 pads per day (60.7%). A positive family history of dysmenorrhea was reported by 48.7%. Figure 1 (see PDF) shows a mean dysmenorrhea score of 24.79 ± 8.63, with 61.3% having moderate, 27.0% severe and 11.7% mild symptoms. Table 2 (see PDF) indicates significant associations of dysmenorrhea severity with family type (p = 0.029), body weight (p = 0.031) and family history (p = 0.015), while other variables were not significant. These findings highlight a high burden of dysmenorrhea and the influence of familial and lifestyle factors.

## Discussion:

The present study highlights a substantial burden of primary dysmenorrhea among rural adolescent girls, with 61.3% reporting moderate pain and 27% reporting severe pain. This prevalence is comparable to other Indian studies where Omidvar *et al.* reported a prevalence of 70.2% among 1,000 college students, with a majority of them experiencing moderate-to-severe pain affecting daily activities [9]. Similarly, Singh *et al.* from Karnataka documented a high frequency of menstrual problems including dysmenorrhea, particularly among undernourished girls with BMI < 18.5 [10]. These findings suggest that our rural sample shares similar risk profiles with adolescent populations across India. Globally, dysmenorrhea affects 50-90% of adolescent girls, as reported by De Sanctis *et al.* [11], confirming that it remains a significant public health issue. One of the key findings of our study was the significant association between BMI categories and severity of dysmenorrhea (p = 0.031). Donayeva *et al.* found that underweight adolescents had significantly higher pain scores than their normal-weight and overweight peers [12]. Similarly, Dhar *et al.* demonstrated that menstrual symptoms were more common among girls with BMI < 18.5 [13]. These associations suggest that low BMI, possibly indicating poor nutrition, hormonal imbalance, or reduced fat stores affecting estrogen and prostaglandin synthesis, may contribute to more severe menstrual cramps. Addressing nutritional status through dietary interventions and health education could therefore reduce the severity of dysmenorrhea. Family history of dysmenorrhea was another strong determinant in our study (p = 0.015). This finding is consistent with Kural *et al.* who reported that girls with a positive family history had significantly increased odds of both occurrence and severity of dysmenorrhea [14]. Meta-analytic evidence further confirms that family history is one of the most reproducible predictors of dysmenorrhea [15]. These findings support the hypothesis that genetic predisposition, shared lifestyle factors and learned pain-coping behaviors within families influence the manifestation of dysmenorrhea in adolescents. Interestingly, girls from nuclear families reported more severe dysmenorrhea compared to those from joint families (p = 0.029). Although this association is less frequently reported in the literature, it may be explained by reduced family support, increased household responsibilities, or lack of shared menstrual health knowledge within nuclear families [16]. Omidvar *et al.* recorded family type in their study, but no significant relationship with severity was reported [17]. Hence, this finding could be context-specific and highlights the need for further qualitative research to explore the psychosocial dimensions of family support during menstruation [18].

On the other hand, certain variables such as age, religion and monthly income, age at menarche, cycle frequency and duration of menstrual flow did not show statistically significant associations with severity in our sample. While Kural *et al.* reported that prolonged bleeding and presence of clots were significantly associated with severe dysmenorrhea [14] and Wang *et al.* highlighted age at menarche and menstrual cycle characteristics as risk factors in their meta-analysis [19], the absence of significant associations in our study may be due to homogeneity of our sample (most girls had menarche ≥12 years and similar cycle durations) or measurement limitations. The high proportion of girls experiencing moderate-to-severe pain has major implications for education and public health. Dysmenorrhea is one of the most common causes of recurrent school absenteeism and its impact on academic performance and psychosocial well-being is well documented [20]. Studies have reported that 20-40% of adolescent girls miss one or more school days per month due to menstrual pain [21]. In our study, many participants reported absenteeism and reduced concentration, further emphasizing the need for school-based health programs. In conclusion, the present study confirms that primary dysmenorrhea is highly prevalent in rural adolescent populations, with low/abnormal BMI, positive family history and nuclear family type being significant determinants of severity. Unlike some studies, we did not find age at menarche, cycle frequency, or duration of flow to be significant. These findings suggest that interventions should focus on improving nutrition, strengthening family and peer support systems and creating awareness through school health programs. Future studies should also explore psychosocial and lifestyle factors in more detail to better understand determinants of severity and develop culturally appropriate, cost-effective strategies to improve adolescent reproductive health and quality of life.
